# Supplementary material for: Transcription-driven DNA supercoiling counteracts H-NS-mediated gene silencing in bacterial chromatin
Source: Nat Commun. 2024 Mar 30;15:2787. doi: 10.1038/s41467-024-47114-w (PMC10981669; doi:10.1038/s41467-024-47114-w)
Supplement: Supplementary file 1 — Supplementary Information [file 41467_2024_47114_MOESM1_ESM.pdf]

Transcription-driven DNA supercoiling counteracts H-NS-mediated gene silencing in bacterial chromatin

Nara Figueroa-Bossi[1], Rocío Fernández-Fernández[2], Patricia Kerboriou[1], Philippe Bouloc[1], Josep Casadesús[3†], María Antonia Sánchez-Romero[2] and Lionello Bossi[1]

[1]*Université Paris-Saclay, CEA, CNRS, Institut de Biologie Intégrative de la Cellule (I2BC), Gif-sur-Yvette, France,* [2]*Departamento de Microbiología y Parasitología, Facultad de Farmacia, Universidad de Sevilla, Sevilla, Spain* and [3]*Departamento de Genética, Facultad de Biología, Universidad de Sevilla, Sevilla, Spain*

† Deceased author

**Corresponding author: lionello.bossi@i2bc.paris-saclay.fr*

Supplementary Information

Supplementary Methods

Construction of relevant alleles.

Strains used in this work were derived from strain MA3409, a derivative of *Salmonella enterica* serovar Typhimurium strain LT2[1] cured for the Gifsy-1 prophage[2]. Generalized transduction was carried out using the high-frequency transducing mutant of phage P22, HT 105/1 *int-201*[3]. Gyrase mutations were introduced by transduction selecting for a linked Tn*10*dCm element[4]. All constructs made by DNA recombineering were obtained in strains harboring λ Red plasmid pKD46[5]. Some of these constructs were described previously[6,7].

Allele Δ[*hilA338*-STM2906]::GFP^{SF}-*kan* (also named *hilA*-GFP^{SF} *kan* ΔK28) replaces the right two-thirds of SPI-1 (the 28 Kb segment extending from position 338 of *hilA* to the end of locus STM2906) with a GFP^{SF}-*kan* cassette, creating a *hilA*-GFP^{SF} translational fusion (see diagram in Supplementary Fig. 1). The 28 Kb deletion was initially made by inserting an FRT-*kan*-FRT cassette amplified from plasmid pKD13[5] with primers AF93 and AF26; subsequent removal of the cassette by the Flp recombinase left a single FRT site that was used to integrate plasmid pMR6 (which carries a single FRT site fused in frame to the GFP^{SF} orf, plus a *kan* gene for selection) in a second Flp-mediated step, as described[8].

Insertions of a *tetR*-P^{tet} cassette replacing material on the left side of SPI-1 were obtained by two-step scarless recombineering[9]. In the first step, the selectable/counter-selectable *tetR* P^{tet} *ccdB-cat* cassette (amplified with primers AE12 and AH30 from plasmid pNNB5[9]) was used to replace the SPI-1 segment extending from the beginning of *sitA* to the *orgA* gene (allele Δ[*sitA-orgA*]::*tetR*-P^{tet}*ccdB-cat*). In the second step, the *ccdB-cat* portion of the construct was replaced by DNA fragments fusing P^{tet} to positions increasingly closer to the *hilD* gene (selecting AHTc-resistance; see Supplementary Figs. 1 and 2). These fragments were generated by "fill-in" PCR of the following oligonucleotide pairs: AG52-AG53 (Δ[*sitA-orgA*]3.0::*tetR*-P^{tet}); AG54-AG55 (Δ[*sitA-prgI*]1.6::*tetR*-P^{tet}); AH34-AH35 (Δ[*sitA-prgH*]1.2::*tetR*-P^{tet}); AH36-AH37 (Δ[*sitA-prgH*]0.8::*tetR*-P^{tet}); AH38-AH39 (Δ[*sitA-prgH*]0.6::*tetR*-P^{tet}).

Constructs analogous to the ones above, but carrying the full *tetRA* cassette (that is, with P^{tet} fused to *tetA*) were constructed using fragments amplified from the chromosomal DNA of strain MA3397 (which carries a Tn*10*dTc insertion in the Gifsy-1 prophage[2]) selecting tetracycline-resistance. Fragments were made with primer pairs AE12-AF20 (Δ[*sitA-orgA*]3.0::*tetRA*); AE12-AF21 (Δ[*sitA-prgI*]1.6::*tetRA*); AE12-AH23 (Δ[*sitA-prgH*]1.2::*tetRA*); AE12-AH24 (Δ[*sitA-prgH*]0.8::*tetRA*); AE12-AH25 (Δ[*sitA-prgH*]0.6::*tetRA*) (Supplemental Figs 1 and 2).

A strain carrying the *tetRA* cassette in the histidine operon attenuator region (*hisL*) immediately upstream of the Rho-independent transcription terminator (T^{hisL}) was constructed using a DNA fragment amplified from MA3397 DNA (see above) with primers AH63 and AH64. This strain (MA14400) was the source of template DNA used to transfer the *tetRA*-T^{hisL} allele to SPI-1. The *tetRA*-T^{hisL} region, amplified with primers AE12-AH67, was transferred by DNA recombineering yielding allele Δ[*sitA-prgH*]1.2::*tetRA*-T^{hisL}. A similar step was followed to insert the *tetRA*-T^{hisL} cassette in the *leu* operon promoter region. For this, MA14400 chromosomal DNA was amplified with primers AM62 and AM63. The resulting fragment was introduced into strain MA13387 and recombinants carrying the cassette at the desired position (*leuL*::*tetRA*-T^{hisL}) were identified. One of the recombinants (strain MA14606) was used in the present study.

A *tetRA* variant with the *tetA* translation initiation codon changed to AAA was constructed by exchanging the *ccdB-cat* segment in strain MA14332 (above) with a fragment amplified from the MA3397 chromosome (above) with primers AH59 (carrying the desired change at the 5' end of the *tetA*-annealing sequence) and AH61 (selecting for AHTc-resistance).

Insertion of the strong gyrase site (SGS) of phage Mu at a position 0.6 Kb upstream of *hilD* TSS was achieved by first inserting the counter-selectable *tetR*-P^{tet}*ccdB-cat* cassette (amplified from

plasmid pNNB5 with primers AG44 and AG45) and subsequently exchanging the cassette with a fragment amplified from plasmid pMP310 (which carries the nuB1 variant of Mu SGS[10]) with primers AO27-AO28. The *tetR*-P^{tet}*tetA*-T^{hisL} cassette was introduced in the SGS background using a recombineering fragment amplified from the MA14400 chromosome with primers AE12-AO31 (see above). Care was taken in designing primer AO31 to ensure that the 3' boundary of the insertion would lie 189 bp closer to *hilD* than the insertion in the SGS-free context to compensate for the SGS addition (see Supplementary Fig. 5).

When required the *tetA* ORF was exchanged with the *cat* ORF using a recombineering fragment amplified from plasmid pKD3[5] with primers AH55 and AN26.

Mutagenesis of the SGS. The segment encompassing the *tetRA*-T^{hisL} cassette and part of the SGS insert of strain MA14726 (*tetR*-P^{tet}*tetA*-T^{hisL} SGS *hilA*-GFP^{SF} *kan* ΔK28) was amplified with primers AO37 (Fw primer, annealing near the end of *tetR*) and AO33 (Rv primer, annealing inside the SGS but with the homology interrupted by a randomized 12-nt stretch - beginning 42 nt from the 5' end of the primer - corresponding to the site of gyrase cleavage). The amplified fragment was introduced into strain MA14733 (*tetR*-P^{tet}*cat*-T^{hisL} SGS *hilA*-GFP^{SF} *kan* ΔK28 / pKD46) and recombinants were selected on tetracycline-supplemented plates. Colonies showing higher green-fluorescence levels (see Supplementary Fig. 5) were picked and used for PCR and Sanger sequence analysis of the mutagenized region. Performing the same experiment with strain MA14733 as the source of template DNA and strain MA14726 / pKD46 as the recombineering host, allowed isolating SGS mutants in the P^{tet}*cat* background.

5' RACE-Seq Experiments. 5'RACE-Seq analysis was conducted on three independent RNA preparations from AHTc-treated and untreated cultures of strains MA14692 (Δ[*sitA*-*prgH*]1.2::*tetRA*-T^{hisL}) and MA14606 (*leuL*::*tetRA*-T^{hisL}). RNA was reverse-transcribed with a mixture of primers AI69 (*hilD*-specific) and AM99 (l*euO*-specifc) in the presence of AI39, the template switching oligonucleotide (TSO). Each of the cDNAs produced (12 samples) was amplified by PCR with primers carrying adapter sequences designed for high throughput sequencing. Oligonucleotide AK47 (which anneals to the TSO sequence) was the common forward primer in all reactions, while a mixture of two oligonucleotides, one annealing in the promoters-proximal region of *hilD*, the other annealing in the promoter-proximal region of *leuO*, and carrying specific index sequences, was used for reverse priming (Supplementary Table 4; amplification program specified in Methods). The PCR products were pooled in equal volumes and the pooled sample was subjected to high throughput sequencing. In the data originating from MA14692, the reads containing the TSO sequence fused to the 5' end of *hilD* were normalized to the reads with the TSO sequence fused to the 5' end of *leuO*. Vice versa for the reads originating for MA14606.

In a separate experiment, the RNA preparations from MA14692 were reverse-transcribed with a mixture of primers AI48 (*prgH*-specific) and AJ33 (*ompA*-specific) in the presence of the TSO. cDNAs was amplified with AJ38 (which anneals to the TSO sequence) as the common forward primer and a single indexed oligonucleotide specific for either *prgH* or *ompA* as the reverse primer. The PCR products were pooled in equal volumes and the pooled sample was subjected to high throughput sequencing. The reads containing the TSO sequence fused to the 5' end of *prgH* were normalized to the reads with the TSO sequence fused to the 5' end of *ompA*.

Supplementary References:

1 Lilleengen, K. Typing of *Salmonella typhimurium* by means of bacteriophage. *Acta Pathol. Microbiol. Scand.* **77**, 2-125 (1948).

2 Figueroa-Bossi, N., Coissac, E., Netter, P. & Bossi, L. Unsuspected prophage-like elements in Salmonella typhimurium. *Mol Microbiol* **25**, 161-173 (1997).

3 Schmieger, H. Phage P22-mutants with increased or decreased transduction abilities. *Mol Gen Genet* **119**, 75-88 (1972).

4 Gari, E. *et al.* A class of gyrase mutants of Salmonella typhimurium show quinolone-like lethality and require rec functions for viability. *Mol Microbiol* **21**, 111-122 (1996).

5 Datsenko, K. A. & Wanner, B. L. One-step inactivation of chromosomal genes in Escherichia coli K-12 using PCR products. *Proc Natl Acad Sci U S A* **97**, 6640-6645 (2000).

6 Bossi, L. *et al.* NusG prevents transcriptional invasion of H-NS-silenced genes. *PLoS Genet* **15**, e1008425, doi:10.1371/journal.pgen.1008425 (2019).

7 Figueroa-Bossi, N. *et al.* Pervasive transcription enhances the accessibility of H-NS-silenced promoters and generates bistability in Salmonella virulence gene expression. *Proc Natl Acad Sci U S A* **119**, e2203011119, doi:10.1073/pnas.2203011119 (2022).

8 Balbontin, R., Ratel, M., Figueroa-Bossi, N. & Bossi, L. Converting an FRT-Tagged Gene into a Fluorescent Protein Gene Fusion by Flp-Mediated Site-Specific Recombination. *Cold Spring Harb Protoc* **2023**, pdb prot107951, doi:10.1101/pdb.prot107951 (2023).

9 Figueroa-Bossi, N., Balbontin, R. & Bossi, L. Scarless DNA Recombineering. *Cold Spring Harb Protoc* **2023**, pdb prot107857, doi:10.1101/pdb.prot107857 (2023).

10 Pato, M. L., Howe, M. M. & Higgins, N. P. A DNA gyrase-binding site at the center of the bacteriophage Mu genome is required for efficient replicative transposition. *Proc Natl Acad Sci U S A* **87**, 8716-8720, doi:10.1073/pnas.87.22.8716 (1990).

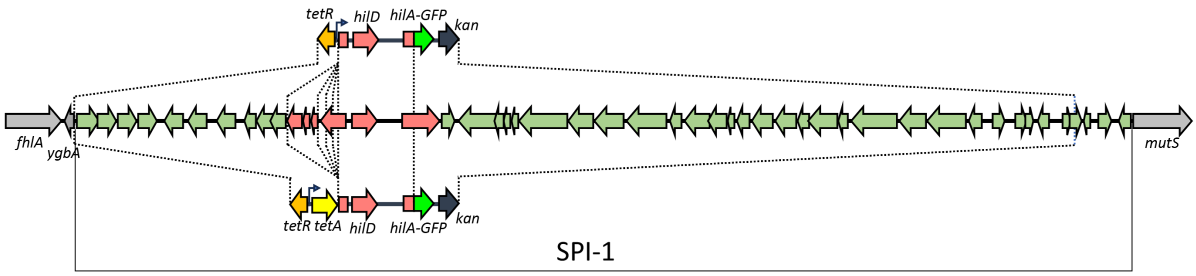

Supplementary Fig. 1 Schematic diagram showing the gene organization in constructs used in this study. Insertion of *tetR*-P^{tet} or *tetR*-P^{tet}-*tetA* cassette removes a DNA segment extending from the left end of SPI-1 to five different postions between 3 Kb and 0.6 Kb upstream of the *hilD* TSS. Insertions of the *hilA*-GFP^{SF}-*kan* cassette removes a 28 Kb portion of SPI-1 extending from inside *hilA* (first 338 bp of the orf) to the end of locus STM2906.

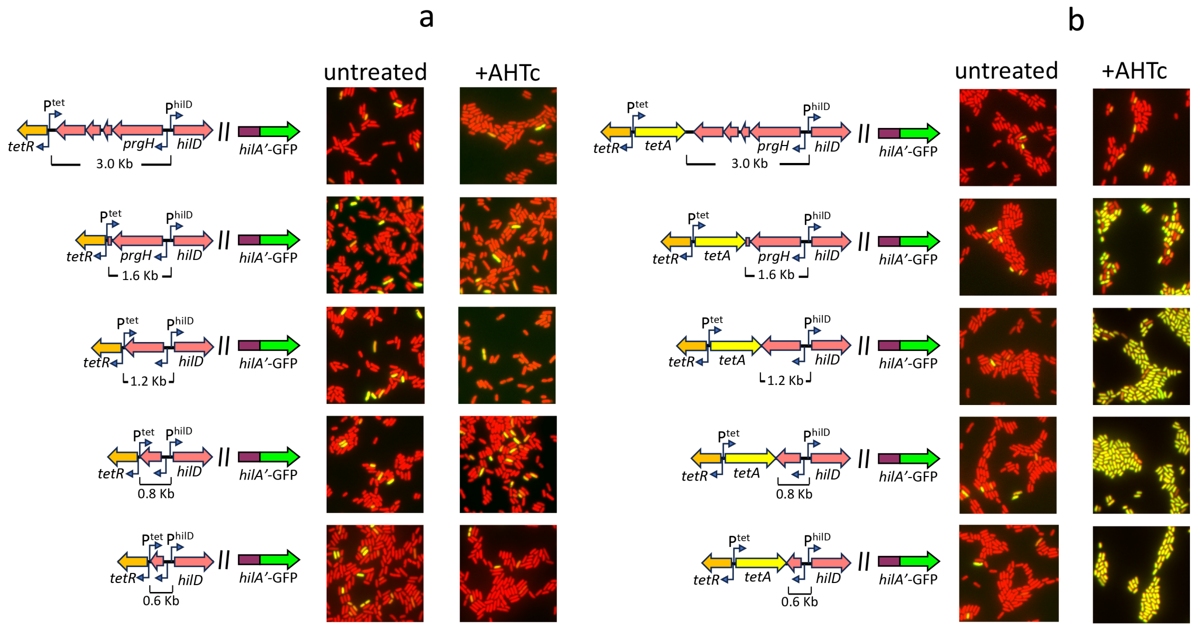

Supplementary Fig. 2 Comparing the effects of activating the P^{tet} promoter in constructs without (a) or with (b) the *tetA* gene fused to P^{tet}. Corresponding pairs of *tetR*-P^{tet} and *tetR*-P^{tet}-*tetA* inserts have identical 3' -flanking sequences. These 3' boundaries fall between 3.0 Kb and 0.6 Kb from the hilD TSS. All strains carry a *hilA*-GFP^{SF} translational fusion and a constitutively expressed mCherry fusion to the P^{tac} promoter. Strains were grown at 37°C to early stationary phase and cells visualized by fluorescence microscopy under 100 x magnification. Representative areas of the microscope field are shown.

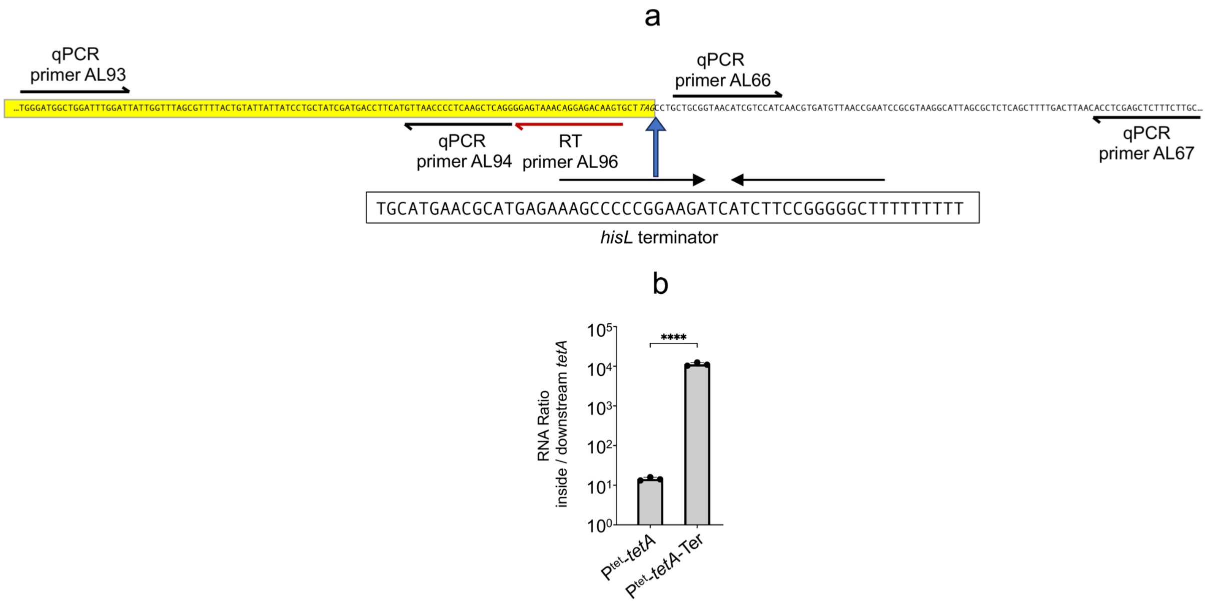

Supplementary Fig. 3 Assessing the efficacy of the *hisL* terminator. a. Sequence of the *tetA*-*prgH* boundary (5'-3') showing the insertion site of the *hisL* terminator (immediately 3' to the *tetA* stop codon; *tetA* sequence shaded in yellow). Also shown are the positions of RT-qPCR primers used for the quantification of the RNA. **b**. Quantification of the RNA gradient. RNA from cultures grown in the presence of AHTc (0.4 µg/ml) was reverse-transcribed in two separate reactions, one with primer AL96 and primer AJ33 (*ompA*-specific, used for normalization), the other with primers AI11 and AJ33 (RT primer AI11, not shown in the diagram, anneals 38 bp downstream of qPCR reverse primer AL67). The cDNAs produced were amplified by qPCR with primer pairs AL93-AL94 and AL66-AL67, respectively. The data shown represent the ratios between the normalized Ct values obtained in the two amplifications in strains MA14694 (P^{tet}-*tetA*) and MA14692 (P^{tet}-*tetA*-Ter). Data originate from n=3 independent experiments and are presented as mean values ±SD. Significance was determined by the unpaired two-tailed Student's t test (****, P ≤ 0.0001).

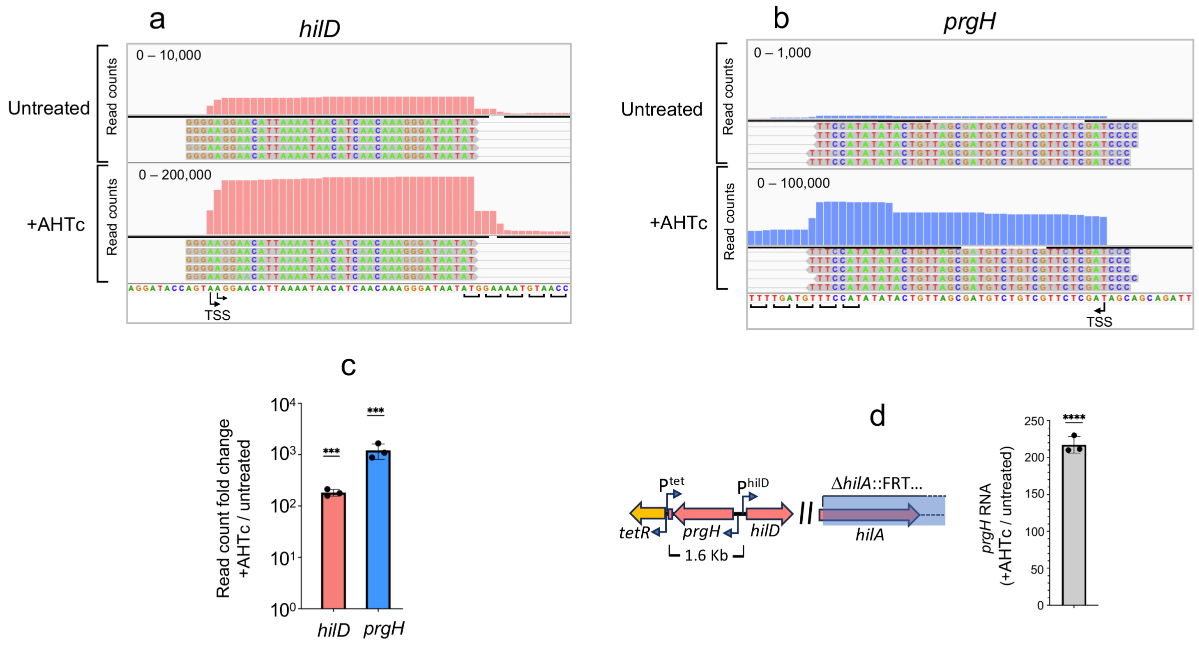

Supplementary Fig. 4 5'RACE-Seq analysis of *hilD* and *prgH* mRNAs. Total RNA was extracted from cultures of strain MA14692 (Δ[*sitA*-*prgH*1.2]::*tetRA*-T^{hisL}) grown at 37°C in the presence or absence of AHTc (0.4 µM). RNA was reverse-transcribed with either a mixture of primers AI69 (*hilD*) and AM99 (*leuO*) (**a**) or AI48 (*prgH*) and AJ33 (*ompA*) (**b**) in the presence of template-switching oligonucleotide (TSO) AI39. The resulting cDNA was subjected to semi-quantitative PCR amplification and high-throughput sequencing as described in Supplementary Methods. Sequence reads were trimmed to remove the TSO sequence up to the terminal 3 Gs. **a** and **b**. Representative Integrative Genomes Viewer (IGV) snapshots of reads in the *hilD* and *prgH* promoter regions. **c**. Semi-quantitative assessment of the abondance of *hilD* and *prgH* mRNA 5' ends. The counts of reads with the TSO sequence fused to *hilD* or *prgH* 5' ends were normalized to the counts of reads in which the TSO was fused to *leuO* and *ompA* 5' ends, respectively. **d**. Quantification of *prgH* RNA as a function of upstream *tetA* transcription in a strain deleted for *hilA*. Strain MA14087 carries a *tetRA* cassette inserted 1.6 Kb upstream of *hilD* (Δ[*orgA*-*prgI*]1.6::*tetR*-P^{tet}*tetA*) and deletion Δ[*hilA5*-STM2906] which removes the SPI-1 segment from the 5th bp of the *hilA* gene to the end of the island. RNA from MA14087 cultures grown in the absence or in the presence of 0.4 µg/ml AHTc was reverse transcribed with primers AI48 (*prgH*) and AJ33 (*ompA*) and the cDNA produced was quantified by qPCR with primer pairs AI50-AI51 (*prgH*) and AJ32-AJ37 (*ompA*). Data in **c** and **d** originate from n=3 independent experiments and are presented as mean values ±SD. Significance was determined by two-tailed ratio-paired t test. P values were 0.0003 (*hilD*) and 0.0007 (*prgH*) in **c** and <0.0001 in **d**. (***, P ≤ 0.001; ****, P ≤ 0.0001).

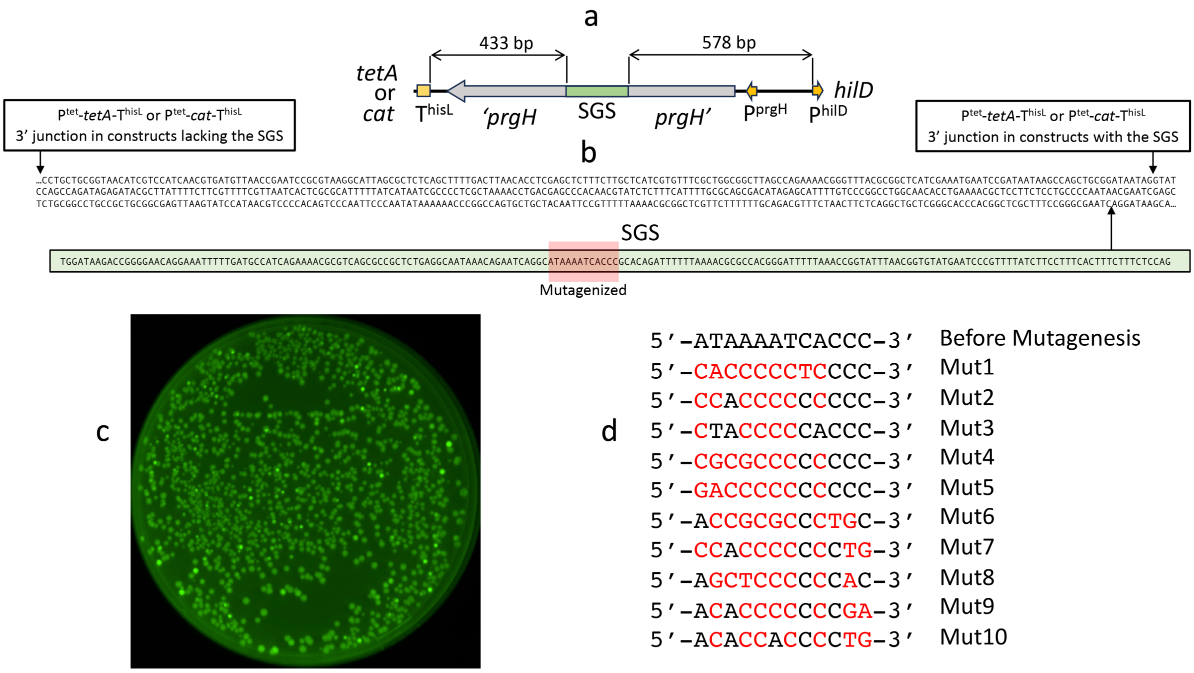

Supplementary Fig. 5 Experiments with the strong gyrase site (SGS). **a**. Structure of the constructs carrying the SGS. **b**. Sequence near the SGS insertion site. The upward-pointing arrow marks the site of insertion of SGS-containing fragment (189 bp, green-shaded box, oriented as it appears in the genome of bacteriophage Mu). Two downward-pointing arrows indicate the position of the 3' boundaries of the P^{tet}*tetA*-Ter insertions. In constructs carrying the SGS insert, this boundary is moved 189 bp downstream to compensate for the SGS addition and maintain the overall distance between the *tetA* cassette and *hilD* unchanged. A 12-bp sequence (encompassing the gyrase cleavage site) subjected to random mutagenesis is shaded in light red. **c**. Representative image of a plate containing colonies from the mutagenic experiment (taken with a Biorad ChemiDoc Touch imaging system under green fluorescence detection conditions). **d**. Sequences of the mutagenized region from 10 clones showing higher fluorescence. Red lettering denotes difference from the original Mu nuB1 sequence[10].

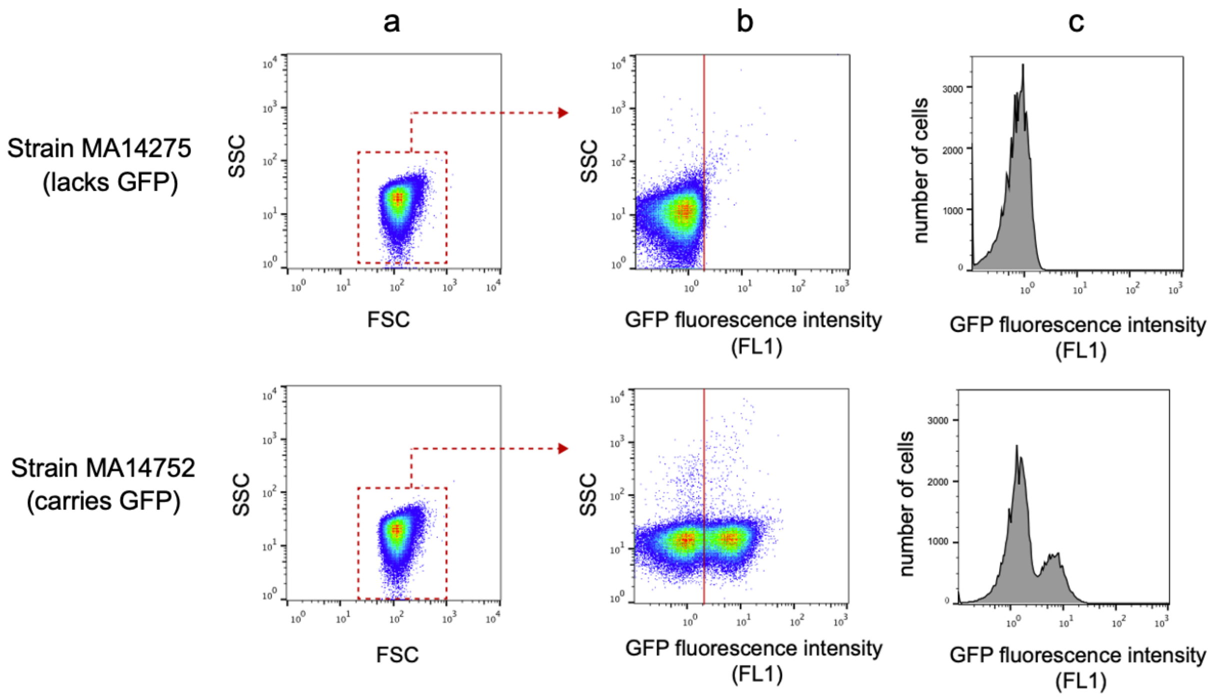

Supplementary Fig. 6 Flow cytometry gating strategy applied in this study. **a**. SSC vs FCS density plot. Each dot or point on the plot represents an individual cell that has passed through the laser beam. The first step was to distinguish bacterial populations of cells based on their forward scatter (FSC) and side scatter (SSC). A gate (red squared box) was applied to identify bacterial cells. **b**. Two-parameter density plot (SSC vs GFP fluorescence intensity). The cells within the gate in **a** were analyzed for GFP expression, using fluorescence (FL1) and side scatter (SSC) as the two measured parameters. **c**. Single-parameter histogram (GFP fluorescence intensity). Data were represented as a histogram where percentage of cells exhibiting the different levels (FL1) of gene expression can be observed. Top row images correspond to MA14275, the control strain (no GFP) used to set the limits of the gates that define the fraction of ON and OFF cells. Lower row images correspond to MA14752, a reporter strain carrying *hilA*-GFP.

Supplementary Table 1. Relevant strains used in this work

Strain	Genotype	Reference
LT2	wild-type	[1]
MA1249	hisC9968::MudJ gyrB1820 zid-6782::Tn10dCm	[4]
MA1564	din-1001::MudJ gyrA208 zej-6790::Tn10dCm	[4]
MA3397	zfh-8157::Tn10dTc sbcE21	[2]
MA3409	Δ[Gifsy-1]	[2]
MA12803	Δ[Gifsy-1] hns-3xFLAG FRT-kan-FRT	[7]
MA13387	Δ[Gifsy-1] leuO-lacZY kan	[6]
MA14067	Δ[Gifsy-1] Δ[Gifsy-2] P^{BAD}gtgR-P^{G2R}nusG-cat Δ[nusG]::aadA hilA338-STM2906]::FRT-kan-FRT	[7]
MA14082	Δ[Gifsy-1] Δ[Gifsy-2] P^{BAD}gtgR-P^{G2R}nusG-cat Δ[nusG]::aadA Δ[hilA338-STM2906]::GFP^{SF}-kan	[7]
MA14087	Δ[Gifsy-1] Δ[Gifsy-2] P^{BAD}gtgR-P^{G2R}nusG-cat Δ[nusG]::aadA Δ[orgA-prgI]1.6::tetR-P^{tet}tetA Δ[hilA5-STM2906]::FRT::pCE40	this work
MA14315	Δ[Gifsy-1] Δ[his]::P^{Tac}mCherry Δ[hilA338-STM2906]::GFP^{SF}-kan	[7]
MA14320	Δ[Gifsy-1] Δ[his]::P^{Tac}mCherry Δ[sitA-orgA]3.0::tetR-P^{tet}tetA Δ[hilA338-STM2906]::GFP^{SF}-kan	this work
MA14321	Δ[Gifsy-1] Δ[his]::P^{Tac}mCherry Δ[sitA-prgI]1.6::tetR-P^{tet}tetA Δ[hilA338-STM2906]::GFP^{SF}-kan	this work
MA14326	Δ[Gifsy-1] Δ[Gifsy-2] P^{BAD}gtgR-P^{G2R}nusG-cat Δ[nusG]::aadA Δ[his]::P^{Tac}mCherry Δ[sitA-prgH]1.2::tetR-P^{tet}tetA Δ[hilA338-STM2906]::GFP^{SF}-kan	this work
MA14327	Δ[Gifsy-1] Δ[Gifsy-2] P^{BAD}gtgR-P^{G2R}nusG-cat Δ[nusG]::aadA Δ[his]::P^{Tac}mCherry Δ[sitA-prgH]0.8::tetR-P^{tet}tetA Δ[hilA338-STM2906]::GFP^{SF}-kan	this work
MA14328	Δ[Gifsy-1] Δ[Gifsy-2] P^{BAD}gtgR-P^{G2R}nusG-cat Δ[nusG]::aadA Δ[his]::P^{Tac}mCherry Δ[sitA-prgH]0.6::tetR-P^{tet}tetA Δ[hilA338-STM2906]::GFP^{SF}-kan	this work
MA14332	Δ[Gifsy-1] Δ[his]::P^{Tac}mCherry Δ[sitA-orgA]3.0::tetR-P^{tet}ccdB cat Δ[hilA338-STM2906]::GFP^{SF}-kan	this work
MA14339	Δ[Gifsy-1] Δ[his]::P^{Tac}mCherry Δ[sitA-prgH]0.6::tetR-P^{tet} Δ[hilA338-STM2906]::GFP^{SF}-kan	this work
MA14340	Δ[Gifsy-1] Δ[his]::P^{Tac}mCherry Δ[sitA-prgH]0.8::tetR-P^{tet} Δ[hilA338-STM2906]::GFP^{SF}-kan	this work
MA14341	Δ[Gifsy-1] Δ[his]::P^{Tac}mCherry Δ[sitA-prgH]1.2::tetR-P^{tet} Δ[hilA338-STM2906]::GFP^{SF}-kan	this work
MA14348	Δ[Gifsy-1] Δ[his]::P^{Tac}mCherry Δ[sitA-orgA]3.0::tetR-P^{tet} Δ[hilA338-STM2906]::GFP^{SF}-kan	this work
MA14349	Δ[Gifsy-1] Δ[his]::P^{Tac}mCherry Δ[sitA-prgI]1.6::tetR-P^{tet} Δ[hilA338-STM2906]::GFP^{SF}-kan	this work
MA14400	Δ[Gifsy-1] hisL::tetR-P^{tet}tetA	this work
MA14403	Δ[Gifsy-1] Δ[his]::P^{Tac}mCherry Δ[sitA-prgH]1.2::tetR-P^{tet}tetA^{AAA} Δ[hilA338-STM2906]::GFP^{SF}-kan	this work
MA14443	Δ[Gifsy-1] hns-3xFLAG FRT Δ[sitA-prgH]1.2::tetR-P^{tet}tetA-T^{hisL}	this work
MA14533	Δ[Gifsy-1] Δ[his]::P^{Tac}mCherry prgH(0.6)::tetR-P^{tet}ccdB cat Δ[hilA338-STM2906]::GFP^{SF}-kan	this work
MA14606	Δ[Gifsy-1] leuL::tetR-P^{tet}tetA-T^{hisL} leuO-lacZY kan	this work
MA14692	Δ[Gifsy-1] Δ[his]::P^{Tac}mCherry Δ[sitA-prgH]1.2::tetR-P^{tet}tetA-T^{hisL} Δ[hilA338-STM2906]::GFP^{SF}-kan	this work
MA14694	Δ[Gifsy-1] Δ[his]::P^{Tac}mCherry Δ[sitA-prgH]1.2::tetR-P^{tet}tetA Δ[hilA338-STM2906]::GFP^{SF}-kan	this work
MA14696	Δ[Gifsy-1] Δ[his]::P^{Tac}mCherry Δ[sitA-prgH]1.2::tetR-P^{tet}cat-T^{hisL} Δ[hilA338-STM2906]::GFP^{SF}-kan	this work
MA14724	Δ[Gifsy-1] Δ[his]::P^{Tac}mCherry prgH(0.6)::SGS(189) Δ[hilA338-STM2906]::GFP^{SF}-kan	this work
MA14726	Δ[Gifsy-1] Δ[his]::P^{Tac}mCherry Δ[sitA-prgH]1.0::tetR-P^{tet}tetA-T^{hisL} prgH(0.6)::SGS(189) Δ[hilA338-STM2906]::GFP^{SF}-kan	this work
MA14752	Δ[Gifsy-1] Δ[his]::P^{Tac}mCherry Δ[sitA-prgH]1.0::tetR-P^{tet}cat-T^{hisL} prgH(0.6)::SGS(189) Δ[hilA338-STM2906]::GFP^{SF}-kan	this work
MA14755	Δ[Gifsy-1] Δ[his]::P^{Tac}mCherry Δ[sitA-prgH]1.0::tetR-P^{tet}cat-T^{hisL} prgH(0.6)::SGS-mut1(189) Δ[hilA338-STM2906]::GFP^{SF}-kan	this work
MA14793	Δ[Gifsy-1] Δ[his]::P^{Tac}mCherry Δ[sitA-prgH]1.0::tetR-P^{tet}tetA-T^{hisL} prgH(0.6)::SGS-mut2(189) Δ[hilA338-STM2906]::GFP^{SF}-kan	this work
MA14804	Δ[Gifsy-1] Δ[his]::P^{Tac}mCherry Δ[sitA-prgH]1.0::tetR-P^{tet}tetA-T^{hisL} prgH(0.6)::SGS(189) gyrA208 zej-6790::Tn10dCm Δ[hilA338-STM2906]::GFP^{SF}-kan	this work
MA14805	Δ[Gifsy-1] Δ[his]::P^{Tac}mCherry Δ[sitA-prgH]1.0::tetR-P^{tet}tetA-T^{hisL} prgH(0.6)::SGS(189) gyrA^{+} zej-6790::Tn10dCm Δ[hilA338-STM2906]::GFP^{SF}-kan	this work
MA14812	Δ[Gifsy-1] Δ[his]::P^{Tac}mCherry Δ[sitA-prgH]1.0::tetR-P^{tet}tetA-T^{hisL} prgH(0.6)::SGS(189) gyrB1820 zid-6782::Tn10dCm Δ[hilA338-STM2906]::GFP^{SF}-kan	this work
MA14813	Δ[Gifsy-1] Δ[his]::P^{Tac}mCherry Δ[sitA-prgH]1.0::tetR-P^{tet}tetA-T^{hisL} prgH(0.6)::SGS(189) gyrB^{+} zid-6782::Tn10dCm Δ[hilA338-STM2906]::GFP^{SF}-kan	this work
MA14888	Δ[Gifsy-1] Δ[Gifsy-2] P^{BAD}gtgR-P^{G2R}nusG-cat Δ[nusG]::aadA Δ[his]::P^{Tac}mCherry Δ[sitA-prgH]1.2::tetR-P^{tet}tetA^{AAA}-T^{hisL} Δ[hilA338-STM2906]::GFP^{SF}-kan	this work

Supplementary Table 2. DNA oligonucleotides used for λ red recombineering

Name	Sequence (5'-3')[a]
AE12	GCAAAGGCTATATTCGATGATTAATTAACCACATTGTTGCGAGTTAAGACCCACTTTCACATTTA
AF20	GGCGGCAAATGAGTTAATACTGGCGGCATGGCGGCTTAAGAACGGCTAAGCACTTGTCTCCTGTTTA
AF21	GATACGGGCGTTGATAATCTACAAACGCAGGTAACAGAGGCGCTGGCTAAGCACTTGTCTCCTGTTTA
AF26	CCGATGGCAAAGCAAAAGTTTAAAATCAACATCGGAGCGGCAGCTGTAGGCTGGAGCTGCTTC
AF93	GTACGGACAGGGCTATCGGTTTAATCGTCCGGTCGTAGTGGTGTCGATCCGTCGACCTGCAGTTC
AG44	CTTCTCAGGCTGCTCGGGCACCCACGGCTCGCTTTCCGGGCGAATCTTAAGACCCACTTTCACATTTA
AG45	CGGTGCAATTAAATACGCCAATACAGGTCGGTGAATTGCTTATCCTGGTCATTGGCATGTTCAA
AG52	CATTGATAGAGTTATTTTACCACTCCCTATCAGTGATAGAGAAAAGTGCCGTTCTTAAGCCG
AG53	ACCCGCCGCCAGGGCGGCGGCAAATGAGTTAATACTGGCGGCATGGCGGCTTAAGAACGGCAC
AG54	CATTGATAGAGTTATTTTACCACTCCCTATCAGTGATAGAGAAAAGTGCCAGCGCCTCTGTTACC
AG55	ACGTCTCAGCAAAATTTGATACGGGCGTTGATAATCTACAAACGCAGGTAACAGAGGCGCTGGC
AH23	GGATTCGGTTAACATCACGTTGATGGACGATGTTACCGCAGCAGGCTAAGCACTTGTCTCCTGTTTA
AH24	GGGCAGGAGAAGGAGCGTTTTCAGGTGTTGCCAGGCCGGGACACTAAGCACTTGTCTCCTGTTTA
AH25	CGGTGCAATTAAATACGCCAATACAGGTCGGTGAATTGCTTATCCTCTAAGCACTTGTCTCCTGTTTA
AH30	GGCGGCAAATGAGTTAATACTGGCGGCATGGCGGCTTAAGAACGGTCATTGGCATGTTCAA
AH34	CATTGATAGAGTTATTTTACCACTCCCTATCAGTGATAGAGAAAAGTGCCTGCTGCG
AH35	GGATTCGGTTAACATCACGTTGATGGACGATGTTACCGCAGCAGGCACTTTTCTC
AH36	CATTGATAGAGTTATTTTACCACTCCCTATCAGTGATAGAGAAAAGTGTCCCGGCC
AH37	GGGGCAGGAGAAGGAGCGTTTTCAGGTGTTGCCAGGCCGGGACACTTTTCTC
AH38	CATTGATAGAGTTATTTTACCACTCCCTATCAGTGATAGAGAAAAGTGGATAAGCAATTCAC
AH39	CGGTGCAATTAAATACGCCAATACAGGTCGGTGAATTGCTTATCCACTTTTCTC
AH55	GAGTTATTTTACCACTCCCTATCAGTGATAGAGAAAAGTGAAATGGAGAAAAAAATCACTGGATATACC
AH59	GATAGAGTTATTTTACCACTCCCTATCAGTGATAGAGAAAAGTGAAAAAAATAGTTCGACAAAGATCG
AH61	CGGTTAACATCACGTTGATGGACGATGTTACCGCAGCAGGCTAAGCACTTGTCTCCTGTTTACTC
AH63	GTGACTGACAATTCATACTATAAATGCGAAATGAAAAAAGCGCTTAAGACCCACTTTCACATTTA
AH64	GGAAGATGATCTTCCGGGGGCTTTCTCATGCGTTCATGCACTAAGCACTTGTCTCCTGTTTA
AH67	GGATTCGGTTAACATCACGTTGATGGACGATGTTACCGCAGCAGGTAACATTCTGCGTTCCTCTTTA
AM62	GCGTTGAGTAGTAGTAGCCCAGTGAAACGAACGATATGTGACATTTAAGACCCACTTTCACATTTAA
AM63	GTACGTATCCGGATATCGTCAACAAAATGCAATGGCGACAGTAACATTCTGCGTTCCTCTTTA
AN26	CCCGGAAGATGATCTTCCGGGGGCTTTCTCATGCGTTCATGCATTACGCCCCGCCCT
AO27	CTAACTTCTCAGGCTGCTCGGGCACCCACGGCTCGCTTTCCGGGCGAATCTGGATAAGACCGGGGAACA
AO28	CGGTGCAATTAAATACGCCAATACAGGTCGGTGAATTGCTTATCCTCTGGAGAAAGAAAGTGAAAGGAAG
AO31	GATTAACGAAAACGAAGAAAATAAGCGTATCTCTATCTGGCTGGATACTAACATTCTGCGTTCCTCTTTA
AO33	CCGGTTTAAAAATCCCGTGGCGCGTTTTAAAAAATCTGTGCNNNNNNNNNNNNGCCTGATTCTGTTTATTGCCTC
AO37	CCGACCTCATTAAGCAGCTCTAATGCGCTGTTAATCACTTTAC

[a] Red lettering denotes sequences annealing to template DNA (plasmid, chromosomal, or primer DNA in fill-in PCR).

Supplementary Table 3. DNA oligonucleotides used as primers for reverse transcription and qPCR

Name	Sequence (5'-3')
AI11	CATTTCGATGAGCCGCGTAA
AI48	ACCGACCTGTATTGGCGTAT
AI50	CGCATCCGTATCCACCTGG
AI51	GACAGGCCGAACACTCTTTG
AI62	TGCCGCAGATAACTTACAGAA
AI63	GTCAGTTTACCGCTCCGAAA
AI69	CAACATCCCAGGTTCGTCAC
AJ32	CGTTGGAGATATTCATGGCGT
AJ33	ACCAGTCGTAGCCCATTTCA
AJ37	GAGACCAGCCCAGTTTAGCA
AL66	CTGCGGTAACATCGTCCATC
AL67	GCAAGAAAGAGCTCGAGGTG
AL93	TGGGATGGCTGGATTTGGAT
AL94	CCTGAGCTTGAGGGGTTAAC
AL96	GCACTTGTCTCCTGTTTACTCC
AM99	CGGCTGAATTCCTCGTCCAT
AN20	GCAAACCACAGCTTCGCAT
AN21	CGACATTCCCAGCGTGTG

Supplementary Table 4. DNA oligonucleotides used as primers for 5' RACE-Seq

Name	Sequence (5'-3')[a]	Anneals to
AI39	GCTAATCATTGCAAGCAGTGGTATCAACGCAGAGTACATrGrGrG (TSO)	
AJ38	AATGATACGGCGACCACCGAGATCTACACTCTTTCCCTACACGACGCTCTTCCGATCTCATTGCAAGCAGTGGTATCAAC	TSO
AK47	AATGATACGGCGACCACCGAGATCTACACTCTTTCCCTACACGACGCTCTTCCGATCTGCAGTGGTATCAACGCAGAG	TSO
AL01	CAAGCAGAAGACGGCATACGAGAAAGGAATGTGACTGGAGTTCAGACGTGTGCTCTTCCGATCTGAGACCAGCCCAGTTTAGCA	ompA
AL02	CAAGCAGAAGACGGCATACGAGAATAGTTGGTGACTGGAGTTCAGACGTGTGCTCTTCCGATCTGAGACCAGCCCAGTTTAGCA	ompA
AL13	CAAGCAGAAGACGGCATACGAGAAAGGAATGTGACTGGAGTTCAGACGTGTGCTCTTCCGATCTCGCATCCGTATCCACCTGG	prgH
AL14	CAAGCAGAAGACGGCATACGAGAATAGTTGGTGACTGGAGTTCAGACGTGTGCTCTTCCGATCTCGCATCCGTATCCACCTGG	prgH
AL17	CAAGCAGAAGACGGCATACGAGAAGTCGTCGTGACTGGAGTTCAGACGTGTGCTCTTCCGATCTGAGACCAGCCCAGTTTAGCA	ompA
AL18	CAAGCAGAAGACGGCATACGAGAACTTCGAGTGACTGGAGTTCAGACGTGTGCTCTTCCGATCTGAGACCAGCCCAGTTTAGCA	ompA
AL39	CAAGCAGAAGACGGCATACGAGAAGTCGTCGTGACTGGAGTTCAGACGTGTGCTCTTCCGATCTCGCATCCGTATCCACCTGG	prgH
AL40	CAAGCAGAAGACGGCATACGAGAACTTCGAGTGACTGGAGTTCAGACGTGTGCTCTTCCGATCTCGCATCCGTATCCACCTGG	prgH
AN72	CAAGCAGAAGACGGCATACGAGAATGGTCAGTGACTGGAGTTCAGACGTGTGCTCTTCCGATCTGCTGCCGGGTATTTGTCAAA	hilD
AN73	CAAGCAGAAGACGGCATACGAGAACACTGTGTGACTGGAGTTCAGACGTGTGCTCTTCCGATCTGCTGCCGGGTATTTGTCAAA	hilD
AN74	CAAGCAGAAGACGGCATACGAGAACTGATCGTGACTGGAGTTCAGACGTGTGCTCTTCCGATCTGCTGCCGGGTATTTGTCAAA	hilD
AN75	CAAGCAGAAGACGGCATACGAGAATTTCACGTGACTGGAGTTCAGACGTGTGCTCTTCCGATCTGCTGCCGGGTATTTGTCAAA	hilD
AN76	CAAGCAGAAGACGGCATACGAGAACCACTCGTGACTGGAGTTCAGACGTGTGCTCTTCCGATCTGCTGCCGGGTATTTGTCAAA	hilD
AN77	CAAGCAGAAGACGGCATACGAGAAATCAGTGTGACTGGAGTTCAGACGTGTGCTCTTCCGATCTGCTGCCGGGTATTTGTCAAA	hilD
AN78	CAAGCAGAAGACGGCATACGAGAAAGGAATGTGACTGGAGTTCAGACGTGTGCTCTTCCGATCTGCTGCCGGGTATTTGTCAAA	hilD
AN79	CAAGCAGAAGACGGCATACGAGAATAGTTGGTGACTGGAGTTCAGACGTGTGCTCTTCCGATCTGCTGCCGGGTATTTGTCAAA	hilD
AN80	CAAGCAGAAGACGGCATACGAGAAAAATGGTGACTGGAGTTCAGACGTGTGCTCTTCCGATCTGCTGCCGGGTATTTGTCAAA	hilD
AN81	CAAGCAGAAGACGGCATACGAGAAGTCGTCGTGACTGGAGTTCAGACGTGTGCTCTTCCGATCTGCTGCCGGGTATTTGTCAAA	hilD
AN82	CAAGCAGAAGACGGCATACGAGAACGATTAGTGACTGGAGTTCAGACGTGTGCTCTTCCGATCTGCTGCCGGGTATTTGTCAAA	hilD
AN83	CAAGCAGAAGACGGCATACGAGAACTTCGAGTGACTGGAGTTCAGACGTGTGCTCTTCCGATCTGCTGCCGGGTATTTGTCAAA	hilD
AN84	CAAGCAGAAGACGGCATACGAGAATGGTCAGTGACTGGAGTTCAGACGTGTGCTCTTCCGATCTACTCCACTGTCACGCTTAAC	hilD
AN85	CAAGCAGAAGACGGCATACGAGAACACTGTGTGACTGGAGTTCAGACGTGTGCTCTTCCGATCTACTCCACTGTCACGCTTAAC	leuO
AN86	CAAGCAGAAGACGGCATACGAGAACTGATCGTGACTGGAGTTCAGACGTGTGCTCTTCCGATCTACTCCACTGTCACGCTTAAC	leuO
AN87	CAAGCAGAAGACGGCATACGAGAATTTCACGTGACTGGAGTTCAGACGTGTGCTCTTCCGATCTACTCCACTGTCACGCTTAAC	leuO
AN88	CAAGCAGAAGACGGCATACGAGAACCACTCGTGACTGGAGTTCAGACGTGTGCTCTTCCGATCTACTCCACTGTCACGCTTAAC	leuO
AN89	CAAGCAGAAGACGGCATACGAGAAATCAGTGTGACTGGAGTTCAGACGTGTGCTCTTCCGATCTACTCCACTGTCACGCTTAAC	leuO
AN90	CAAGCAGAAGACGGCATACGAGAAAGGAATGTGACTGGAGTTCAGACGTGTGCTCTTCCGATCTACTCCACTGTCACGCTTAAC	leuO
AN91	CAAGCAGAAGACGGCATACGAGAATAGTTGGTGACTGGAGTTCAGACGTGTGCTCTTCCGATCTACTCCACTGTCACGCTTAAC	leuO
AN92	CAAGCAGAAGACGGCATACGAGAAAAATGGTGACTGGAGTTCAGACGTGTGCTCTTCCGATCTACTCCACTGTCACGCTTAAC	leuO
AN93	CAAGCAGAAGACGGCATACGAGAAGTCGTCGTGACTGGAGTTCAGACGTGTGCTCTTCCGATCTACTCCACTGTCACGCTTAAC	leuO
AN94	CAAGCAGAAGACGGCATACGAGAACGATTAGTGACTGGAGTTCAGACGTGTGCTCTTCCGATCTACTCCACTGTCACGCTTAAC	leuO
AN95	CAAGCAGAAGACGGCATACGAGAACTTCGAGTGACTGGAGTTCAGACGTGTGCTCTTCCGATCTACTCCACTGTCACGCTTAAC	leuO

[a] Red lettering denotes PCR priming sequences. Colored hexameric sequences denote indexes used in Illumina sequencing.
