# Supplementary material for: Transcription-driven DNA supercoiling counteracts H-NS-mediated gene silencing in bacterial chromatin
Source: Nat Commun. 2024 Mar 30;15:2787. doi: 10.1038/s41467-024-47114-w (PMC10981669; doi:10.1038/s41467-024-47114-w)
Supplement: Supplementary file 3 — Reporting Summary [file 41467_2024_47114_MOESM3_ESM.pdf]

nature portfolio

Corresponding author(s): Lionello Bossi

Last updated by author(s): Mar 3, 2024

Reporting Summary

Nature Portfolio wishes to improve the reproducibility of the work that we publish. This form provides structure for consistency and transparency in reporting. For further information on Nature Portfolio policies, see our Editorial Policies and the Editorial Policy Checklist.

Statistics

For all statistical analyses, confirm that the following items are present in the figure legend, table legend, main text, or Methods section.

n/a	Confirmed	
☐	☒	The exact sample size (*n*) for each experimental group/condition, given as a discrete number and unit of measurement
☐	☒	A statement on whether measurements were taken from distinct samples or whether the same sample was measured repeatedly
☐	☒	The statistical test(s) used AND whether they are one- or two-sided<br>*Only common tests should be described solely by name; describe more complex techniques in the Methods section.*
☒	☐	A description of all covariates tested
☐	☒	A description of any assumptions or corrections, such as tests of normality and adjustment for multiple comparisons
☐	☒	A full description of the statistical parameters including central tendency (e.g. means) or other basic estimates (e.g. regression coefficient) AND variation (e.g. standard deviation) or associated estimates of uncertainty (e.g. confidence intervals)
☐	☒	For null hypothesis testing, the test statistic (e.g. *F*, *t*, *r*) with confidence intervals, effect sizes, degrees of freedom and *P* value noted<br>*Give P values as exact values whenever suitable.*
☒	☐	For Bayesian analysis, information on the choice of priors and Markov chain Monte Carlo settings
☒	☐	For hierarchical and complex designs, identification of the appropriate level for tests and full reporting of outcomes
☒	☐	Estimates of effect sizes (e.g. Cohen's *d*, Pearson's *r*), indicating how they were calculated

Our web collection on statistics for biologists contains articles on many of the points above.

Software and code

Policy information about availability of computer code

Data collection	Data acquisition for RT-qPCR analyses was carried out using a LightCycler 480 system software. Flow cytometry data acquisition was with a Cytomics FL500 MPL cytometer from Beckman Coulter Inc. and analyzed with FlowJo v8.7 Software (BD Life Sciences).
Data analysis	GraphPad Prism version 9.5.1 (used to measured statistical significance in RT-qPCR measurements and flow cytometry analysis). Primer3 (Untergasser et al; Nucleic Acids Res 40:e115, 2012; used for PCR primer design); Bcl2fastq2 V2.2.18.12 program (demultiplexing); Cutadapt 1.15 (adapter trimming); BWA 0.6.2-r126 (mapping reads to the Salmonella genome); Bedtools genomecov (generating bedgraph files). BedGraphToBigWig command line utility from UCSC (converting coverage track (to the BigWig format); Samtools view tool of the Samtools suite (ChIP-Seq experiments; measuring read counts in selected regions of the Salmonella chromosome); Integrative Genome Viewer (IGV; to visualize Bam and BigWig files).

For manuscripts utilizing custom algorithms or software that are central to the research but not yet described in published literature, software must be made available to editors and reviewers. We strongly encourage code deposition in a community repository (e.g. GitHub). See the Nature Portfolio guidelines for submitting code & software for further information.

Data

Policy information about availability of data

All manuscripts must include a data availability statement. This statement should provide the following information, where applicable:

- Accession codes, unique identifiers, or web links for publicly available datasets
- A description of any restrictions on data availability
- For clinical datasets or third party data, please ensure that the statement adheres to our policy

The ChiP-Seq data generated in this study have been deposited in the ArrayExpress database under the accession code E-MTAB-13436 [https://www.ebi.ac.uk/biostudies/arrayexpress/studies/E-MTAB-13436?key=6a825b05-79a1-4cad-b908-45cbc5cd1e89]. The FASTA and gff3 files of Salmonella enterica serovar Typhimurium strain MA14443 used in the ChiP-Seq analysis are provided in the Supplementary Information/Source Data file. The 5’RACE-Seq data in Fig. 3c and Supplementary Fig. 4a have been deposited in the ArrayExpress database under the accession code E-MTAB-13482 [https://www.ebi.ac.uk/biostudies/arrayexpress/studies/E-MTAB-13482?key=2261e0c5-5608-4102-a9e6-ff08f9ba7fe8]. The 5’RACE-Seq data in Supplementary Fig. 4b are provided in the the Supplementary Information/Source Data file. The density plots from the flow cytometry analysis in Fig. 1 a,b,c,d; Fig. 4 a,d and Fig. 5 a,c are provided in the the Supplementary Information/Source Data file. The raw data from the experiments in Fig. 1e,f,g; Fig.2b; Fig. 3b,d; Fig. 4b,c,e,f; Fig. 5b,d; Supplementary Fig. 3b, and Supplementary Fig. 4c,d are provided in the the Supplementary Information/Source Data file.

Research involving human participants, their data, or biological material

Policy information about studies with human participants or human data. See also policy information about sex, gender (identity/presentation), and sexual orientation and race, ethnicity and racism.

Reporting on sex and gender	N/A
Reporting on race, ethnicity, or other socially relevant groupings	N/A
Population characteristics	N/A
Recruitment	N/A
Ethics oversight	N/A

Note that full information on the approval of the study protocol must also be provided in the manuscript.

Field-specific reporting

Please select the one below that is the best fit for your research. If you are not sure, read the appropriate sections before making your selection.

[x] Life sciences [ ] Behavioural & social sciences [ ] Ecological, evolutionary & environmental sciences

For a reference copy of the document with all sections, see nature.com/documents/nr-reporting-summary-flat.pdf

Life sciences study design

All studies must disclose on these points even when the disclosure is negative.

Sample size	All experiments described here were performed on bacterial cultures inoculated from single colonies. For each strain, experiments were conducted three to five times, as indicated in the figure legends, on different days, each time originating from a distinct single colony. The decision on the number of replicates was made based on established experimental protocols in this field, without prior calculation of sample size. A typical sample size of n=3 to n=5 was chosen to ensure adequate statistical power and to reduce random variations, in line with prevailing practices in the field.
Data exclusions	No data were excluded.
Replication	Reproducibility was ensured by at least 3 independent replicates. All replication attempts were successful.
Randomization	Bacterial cultures were derived from randomly selected colonies on Petri dishes, ensuring the absence of bias in sample selection. All cultures were grown under identical conditions, eliminating the need to control covariates. This methodology represents standard practice in our field and guarantees experimental reproducibility.
Blinding	N/A because of what described above.

Reporting for specific materials, systems and methods

We require information from authors about some types of materials, experimental systems and methods used in many studies. Here, indicate whether each material, system or method listed is relevant to your study. If you are not sure if a list item applies to your research, read the appropriate section before selecting a response.

Materials & experimental systems

n/a	Involved in the study
☒	☐ Antibodies
☒	☐ Eukaryotic cell lines
☒	☐ Palaeontology and archaeology
☒	☐ Animals and other organisms
☒	☐ Clinical data
☒	☐ Dual use research of concern
☒	☐ Plants

Methods

n/a	Involved in the study
☐	☒ ChIP-seq
☐	☒ Flow cytometry
☒	☐ MRI-based neuroimaging

Plants

Seed stocks: N/A

Novel plant genotypes: N/A

Authentication: N/A

ChIP-seq

Data deposition

☒ Confirm that both raw and final processed data have been deposited in a public database such as GEO.

☒ Confirm that you have deposited or provided access to graph files (e.g. BED files) for the called peaks.

Data access links
May remain private before publication.

The ChiP-Seq data generated in this study have been deposited in the ArrayExpress database under the accession code E-MTAB-13436 [https://www.ebi.ac.uk/biostudies/arrayexpress/studies/E-MTAB-13436?key=6a825b05-79a1-4cad-b908-45cbc5cd1e89]. Processed data are provided at: https://drive.google.com/drive/folders/1f8jeLwPA3sj0T38hSeicofzgG_qsZ81S?usp=sharing.

Files in database submission

Input_untreated_1_R1_cutadapt.fastq.gz
Input_untreated_1_R2_cutadapt.fastq.gz
Input_untreated_2_R1_cutadapt.fastq.gz
Input_untreated_2_R2_cutadapt.fastq.gz
Input_untreated_3_R1_cutadapt.fastq.gz
Input_untreated_3_R2_cutadapt.fastq.gz
Input_AHTc_1_R1_cutadapt.fastq.gz
Input_AHTc_1_R2_cutadapt.fastq.gz
Input_AHTc_2_R1_cutadapt.fastq.gz
Input_AHTc_2_R2_cutadapt.fastq.gz
Input_AHTc_3_R1_cutadapt.fastq.gz
Input_AHTc_3_R2_cutadapt.fastq.gz
ChIP_untreated_1_R1_cutadapt.fastq.gz
ChIP_untreated_1_R2_cutadapt.fastq.gz
ChIP_untreated_2_R1_cutadapt.fastq.gz
ChIP_untreated_2_R2_cutadapt.fastq.gz
ChIP_untreated_3_R1_cutadapt.fastq.gz
ChIP_untreated_3_R2_cutadapt.fastq.gz
ChIP_AHTc_1_R1_cutadapt.fastq.gz
ChIP_AHTc_1_R2_cutadapt.fastq.gz
ChIP_AHTc_2_R1_cutadapt.fastq.gz
ChIP_AHTc_2_R2_cutadapt.fastq.gz
ChIP_AHTc_3_R1_cutadapt.fastq.gz
ChIP_AHTc_3_R2_cutadapt.fastq.gz
Input_1_untreated_sorted.bam

Input_1_untreated_sorted.bam.bai
Input_2_untreated_sorted.bam
Input_2_untreated_sorted.bam.bai
Input_3_untreated_sorted.bam
Input_3_untreated_sorted.bam.bai
Input_1_AHTc_sorted.bam
Input_1_AHTc_sorted.bam.bai
Input_2_AHTc_sorted.bam
Input_2_AHTc_sorted.bam.bai
Input_3_AHTc_sorted.bam
Input_3_AHTc_sorted.bam.bai
ChIP_untreated_1_sorted.bam
ChIP_untreated_1_sorted.bam.bai
ChIP_untreated_2_sorted.bam
ChIP_untreated_2_sorted.bam.bai
ChIP_untreated_3_sorted.bam
ChIP_untreated_3_sorted.bam.bai
ChIP_AHTc_1_sorted.bam
ChIP_AHTc_1_sorted.bam.bai
ChIP_AHTc_2_sorted.bam
ChIP_AHTc_2_sorted.bam.bai
ChIP_AHTc_3_sorted.bam
ChIP_AHTc_3_sorted.bam.bai

Genome browser session
(e.g. UCSC)

FASTA and gff3 files of the genome of Salmonella strain MA14443 used in the CHIP-Seq analysis are provided in the the Supplementary Information/Source Data file.

Methodology

Replicates

Three independent cultures of strain MA14443 grown in LB (untreated_input_1, _ 2, _3 and untreated_ChIP_1,_ 2, _3) and in LB supplemented to AHTc (AHTc_input_1, _ 2, _3 and AHTc_ChIP_1,_ 2, _3).

Sequencing depth

Number of sequencing cycles: 75 (Paired end)
Sequencing kit: NextSeq 500/550 Mid Output Kit v2 (150 cycles)
Untreated_Input_1, total reads: 23,999,778
Untreated_Input_2, total reads: 25,847,008
Untreated_Input_3, total reads: 20,616,812
Untreated_ChIP_1, total reads: 13,983,372
Untreated_ChIP_2, total reads: 23,806,746
Untreated_ChIP_3, total reads: 20,616,812
AHTc_Input_1, total reads: 20,148,382
AHTc_Input_2, total reads: 23,033,520
AHTc_Input_3, total reads: 23,707,504
AHTc_ChIP_1, total reads: 23,045,190
AHTc_ChIP_2, total reads: 21,547,530
AHTc_ChIP_3, total reads: 24,351,294

Antibodies

ANTI-FLAG® M2 Affinity Gel. A2220.

Peak calling parameters

The analysis described in this paper focuses on a tiny portion of the Salmonella genome: the 44 KB Pathogenicity Island 1. Peaks were visualized by eye and the read counts in peak areas were were calculated using the Samtools view tool of the Samtools suite.

Data quality

Final libraries quality was assessed on an Agilent Bioanalyzer 2100, using an Agilent High Sensitivity DNA Kit. Libraries were pooled in equimolar proportions and sequenced on a Paired-End 2x75 bp run, on an Illumina NextSeq500 instrument.

Software

Demultiplexing of raw data from the Illumina sequencer was performed with the bcl2fastq2 V2.2.18.12 program and adapters were trimmed with Cutadapt1.15. The reads from the CHIP- Seq experiments were mapped on the genomes of Salmonella enterica serovar Typhimurium strain MA14443 with BWA 0.6.2-r126. Bedgraph files were generated from aligned Bam files using bedtools genomecov. Coverage track (number of reads per base) was converted to the BigWig format using the bedGraphToBigWig command line utility from UCSC.

Flow Cytometry

Plots

Confirm that:

- [x] The axis labels state the marker and fluorochrome used (e.g. CD4-FITC).
- [x] The axis scales are clearly visible. Include numbers along axes only for bottom left plot of group (a 'group' is an analysis of identical markers).
- [x] All plots are contour plots with outliers or pseudocolor plots.
- [x] A numerical value for number of cells or percentage (with statistics) is provided.

Methodology

Sample preparation	Bacterial cultures were grown at 37 ºC in LB with or without AHTc until an OD600 of 0.7-0.8. Cells were washed and diluted in Phosphate Buffered Saline (PBS) to a final concentration of ~10^7 cells/ml. Data were collected for 100,000 events per sample and were analyzed with FlowJo v8.7 Software (BD Life Sciences). Data are represented either by a dot plot (side scatter [cell size] vs fluorescence intensity) or histograms (% cells count vs fluorescence intensity).
Instrument	Data acquisition and analysis were performed using a Cytomics FL500 MPL cytometer rom Beckman Coulter Inc. Serial number: AL010002.
Software	Software for data acquisition was MXP Cytometer 2.2 (supplied for general use with FC500 MPL cytometers). Software for data analysis was FlowJo X 10.0.7r with Mac OS X as the Operating System
Cell population abundance	Fluorescence of all bacterial cells in the cultures was analysed. No sorting of cells was made as the goal of the experiment was to examine the whole cell population.
Gating strategy	A 2D histogram of side scatter (SSC) vs. forward scatter (FSC) was used to distinguishi events from the bacterial cell population from those of background noise. The threshold value for FSC was set to 3. Bacterial cell population was gated in this 2D histogram for subsequent measure of GFP fluorescence intensity. Fluorescence values for 100,000 events were compared with the data from the reporter-less control strain, thus yielding the fraction of ON and OFF cells

- [x] Tick this box to confirm that a figure exemplifying the gating strategy is provided in the Supplementary Information.
