# Supplementary figures and images for: Transcription-driven DNA supercoiling counteracts H-NS-mediated gene silencing in bacterial chromatin
Source: Nat Commun. 2024 Mar 30;15:2787. doi: 10.1038/s41467-024-47114-w (PMC10981669; doi:10.1038/s41467-024-47114-w)

Figure 1a

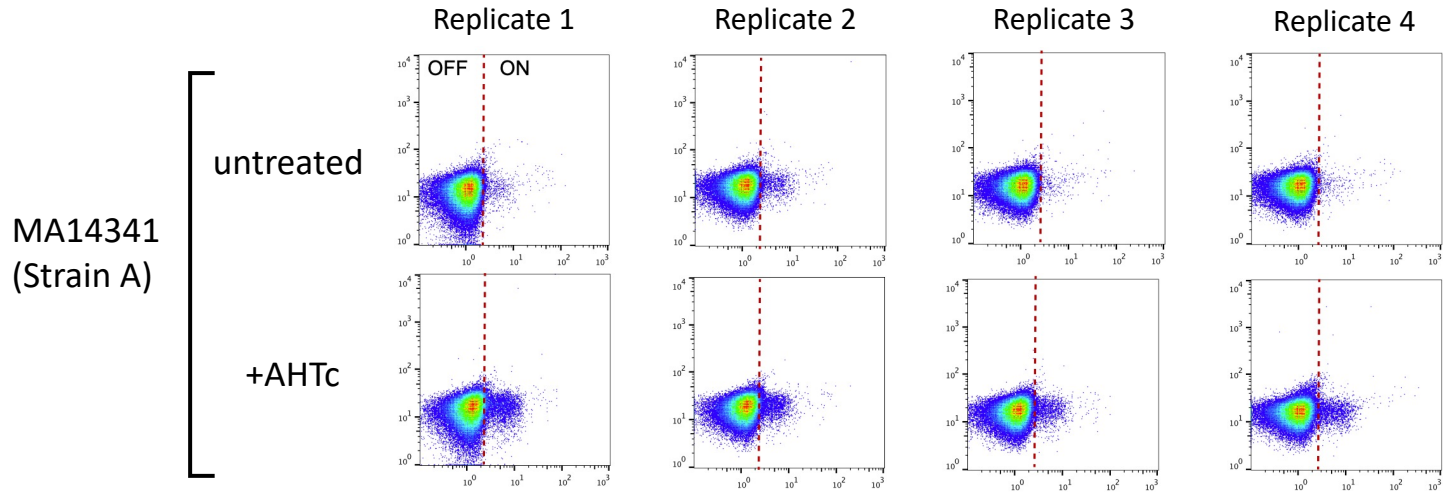

Figure 1b

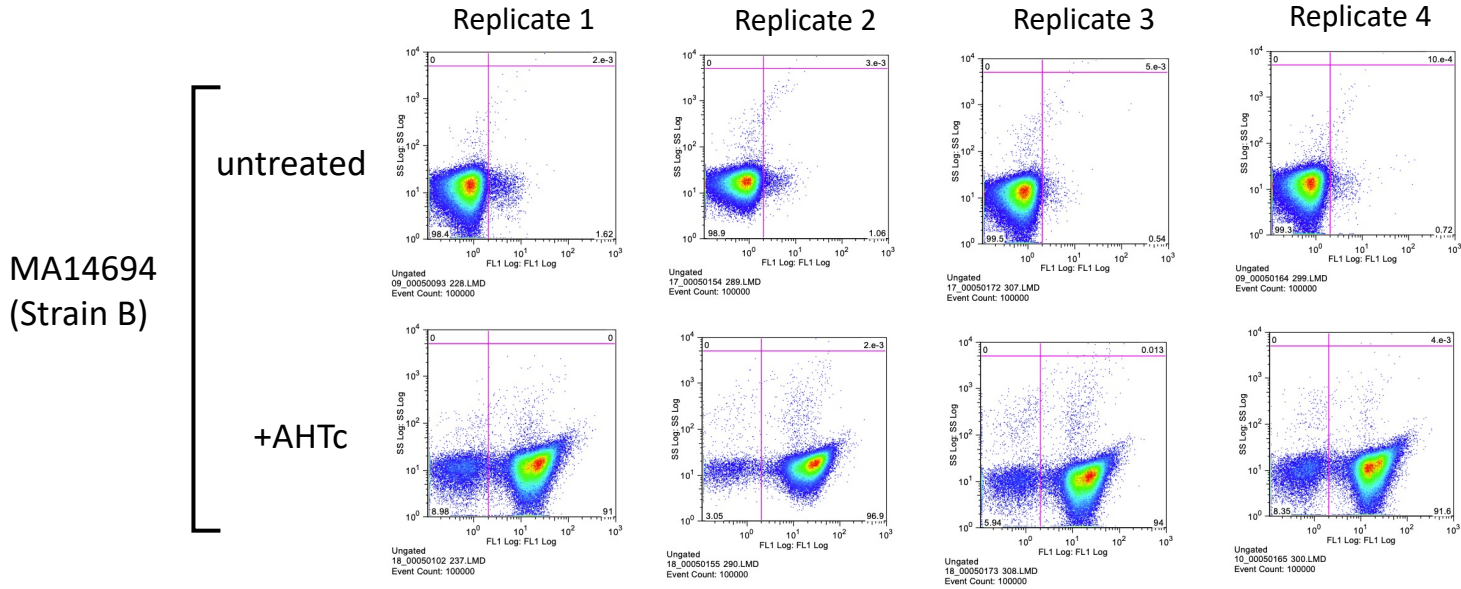

Figure 1c

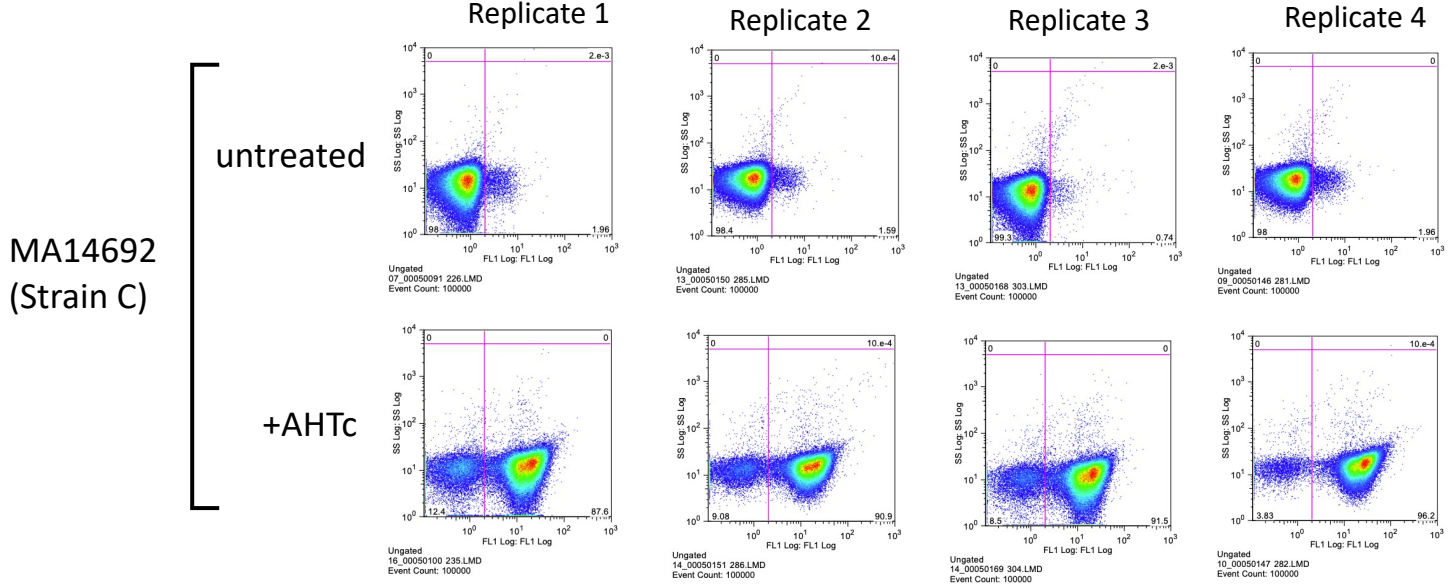

Figure 1d

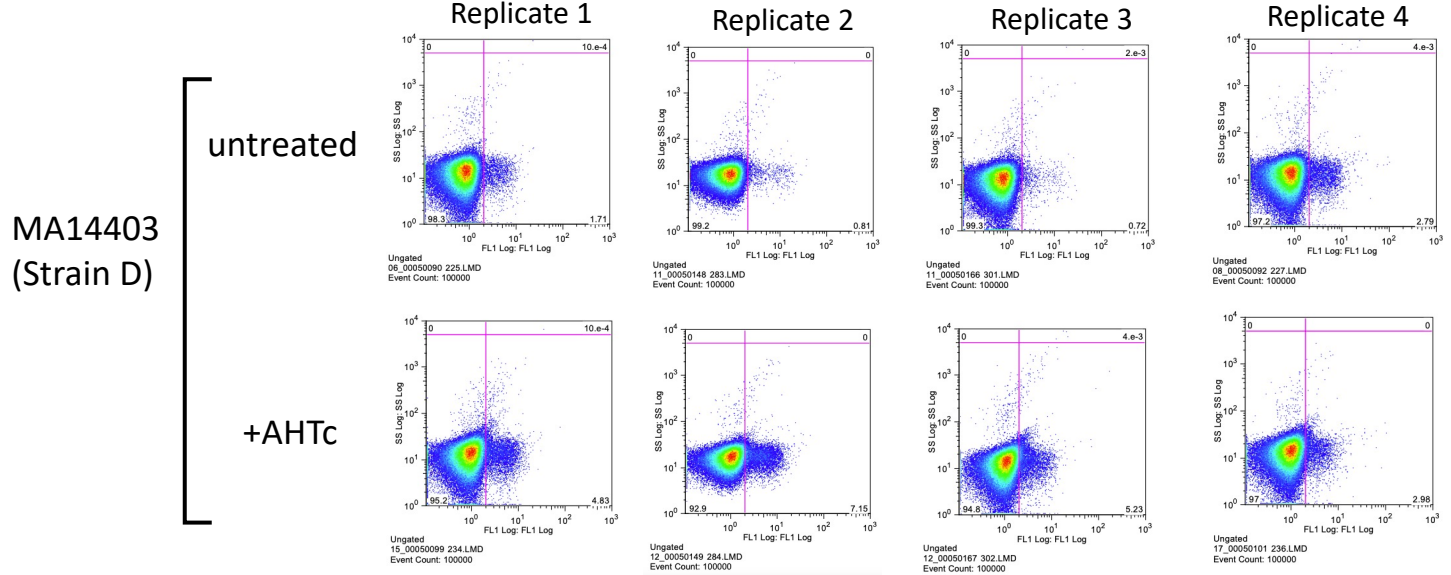

Figure 4a

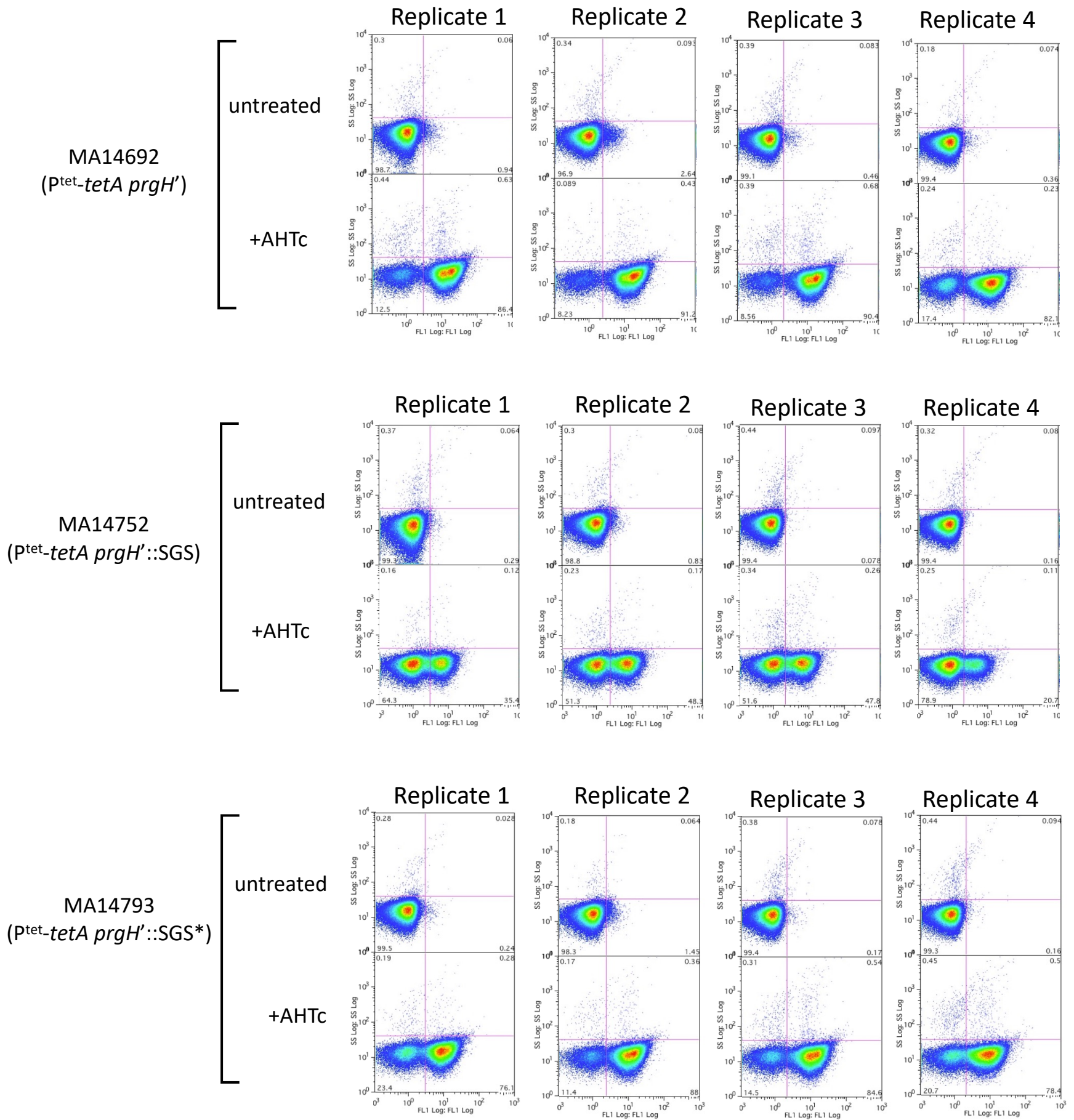

Figure 4d

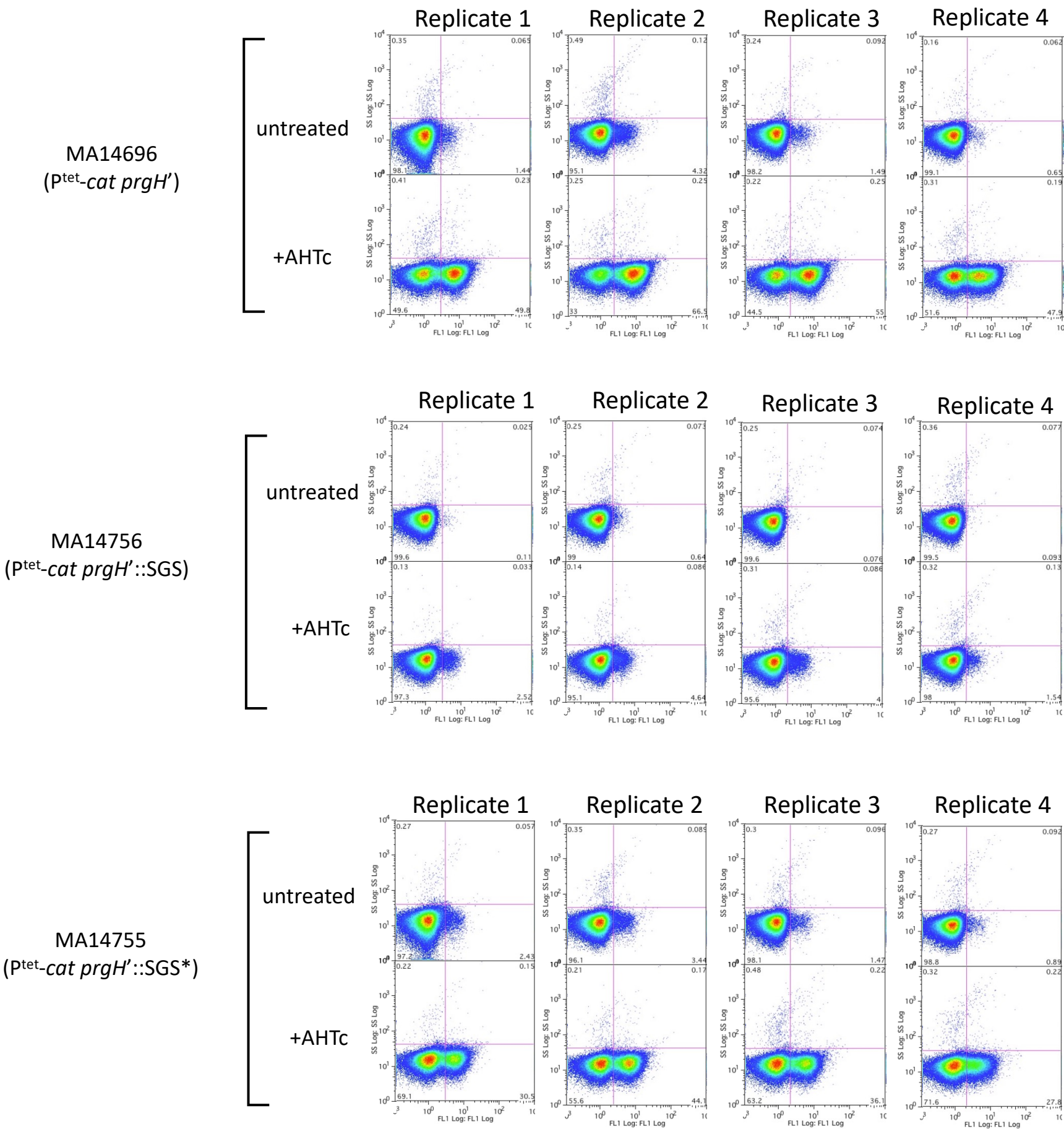

Figure 5a

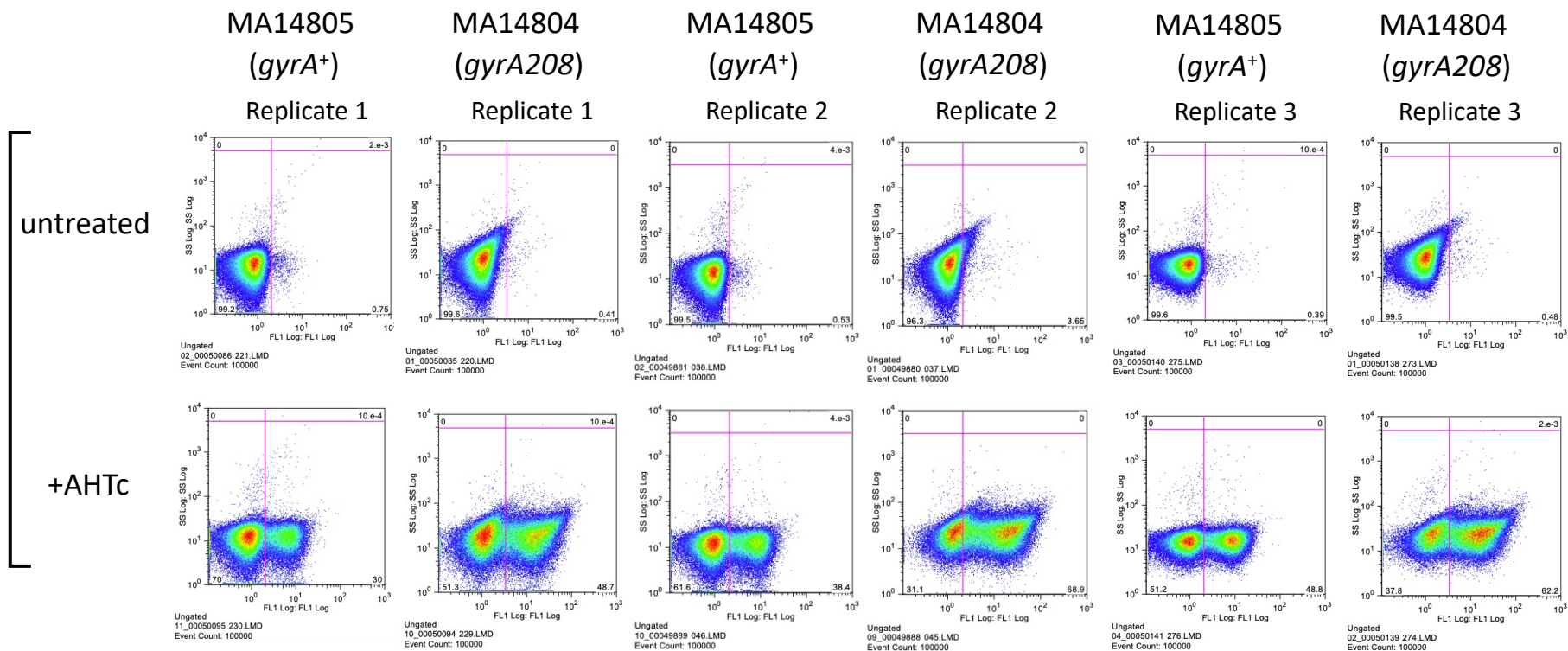

Figure 5c

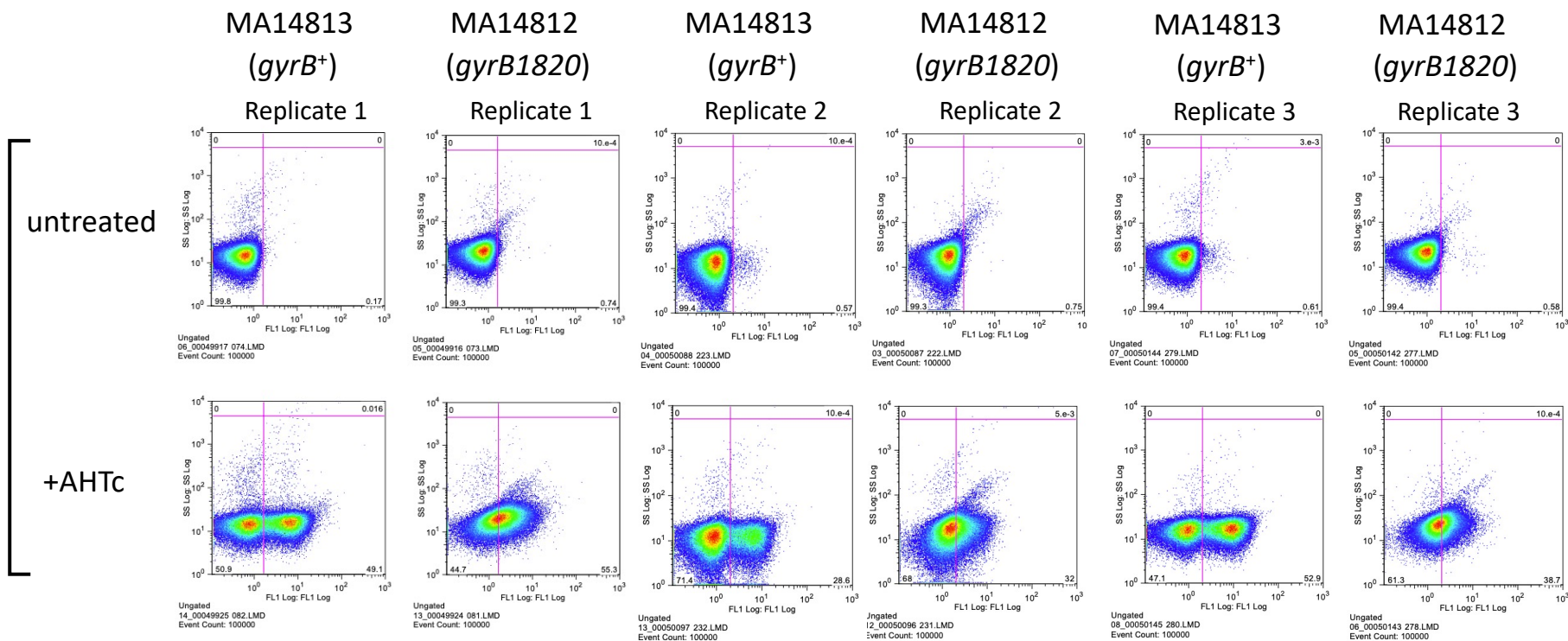

Supplement: Supplementary file 4 — Source Data [file 41467_2024_47114_MOESM4_ESM.zip › Source data/Flow Cytometry Density Plots.pdf]
